# In this month’s Bulletin

**DOI:** 10.2471/BLT.20.001120

**Published:** 2020-11-01

This month’s special issue is on primary health care, introduced in an editorial by Tedros Adhanom Ghebreyesus (726). Shannon Barkley et al. (727) discuss what’s needed to achieve the vision of primary health care and Teri A Reynolds et al. (728) describe the specifics of emergency, critical and operative care components.

Gary Humphreys (731–732) reports on the use of digital health services during the coronavirus pandemic and talks to Angela Nguku (733–734) about the vital importance of community involvement in primary health care.

## Afghanistan, Albania, Angola, Armenia, Bangladesh, Benin, Burkina Faso, Burundi, Cambodia, Cameroon, Chad, Comoros, Congo, Côte d’Ivoire, Democratic Republic of the Congo, Dominican Republic, Egypt, Ethiopia, Gabon, Gambia, Ghana, Guatemala, Guinea, Haiti, Honduras, India, Indonesia, Jordan, Kenya, Kyrgyzstan, Lesotho, Liberia, Malawi, Maldives, Mali, Mozambique, Myanmar, Namibia, Nepal, Nigeria, Pakistan, Papua New Guinea, Peru, Philippines, Rwanda, Senegal, Sierra Leone, South Africa, Tajikistan, Timor-Leste, Togo, Uganda, United Republic of Tanzania, Yemen, Zambia, Zimbabwe

### Where to provide primary care?

Catherine Arsenault et al. (735–746) examine the quality of hospital-based services.

## Bangladesh, Bhutan, Democratic People's Republic of Korea, India, Indonesia, Maldives, Myanmar, Nepal, Sri Lanka, Thailand, Timor-Leste

### How to keep a rural health workforce.

Tomas Zapata et al. (815–817) detail lessons of effective strategies.

## India

### How to measure quality of care?

Devaki Nambiar et al. (747–753) field-test indicators.

### Facets of primary health care

Luke N Allen et al. (754–765) review the evidence on social determinants of noncommunicable diseases. 

Eliana E Kim et al. (766–772) argue that essential surgery should be provided by family physicians.

Emma Sacks et al. (773–780) explain the link between community involvement and universal health coverage.

Etienne V Langlois et al. (781–791) count measures needed to strengthen care provision in low- and middle-income countries.

Somtanuek Chotchoungchatchai et al. (792–800) analyse contributions to the sustainable development goals.

Kumanan Rasanathan & Tim G Evans (801–808) recall the Declaration of Astana.

Arit Udoh et al. (809–811) detail a professional development plan for the pharmacy staff.

Jan De Maeseneer et al. (812–814) introduce a campaign for reassignment of 30% of health system funding.

Sowmya Kadandale et al. (818–820) uncover disconnects between primary care and climate policies.

